# Comparison of laparoscope-assisted single-needle laparoscopic percutaneous extraperitoneal closure versus open repair for pediatric inguinal hernia

**DOI:** 10.1186/s12893-022-01787-6

**Published:** 2022-09-09

**Authors:** Shaofeng Wu, Xiaoyu Xing, Rong He, Haiteng Zhao, Liang Zhong, Jie Sun

**Affiliations:** Department of Urology, Shanghai Children’s Medical Center, School of Medicine, Shanghai Jiaotong University, 1678 Dongfang Rd, Pudong District, Shanghai, 200127 China

**Keywords:** Inguinal hernia, Patent processus vaginalis, Laparoscopic hernia repair, Minimally invasive surgery, Children

## Abstract

**Background:**

Laparoscopic-assisted repairs for pediatric inguinal hernia have gained gradual acceptance over the past decade. However, consensus about the optimal management is still lacking. The aim of this study is to compare outcomes of a modified laparoscope-assisted single-needle laparoscopic percutaneous extraperitoneal closure (LPEC) versus open repair of pediatric hernias/hydrocele in a single institution.

**Materials and methods:**

We retrospectively reviewed the medical data of children who underwent laparoscope-assisted single-needle LPEC and open repair (OR) for inguinal hernia from 2014 to 2019. Data collection included demographics, laterality of hernia, surgical time and time to follow-up. We also reviewed and analyzed the evidence of recurrence, the incidence of metachronous contralateral inguinal hernia (MCIH), and other complications.

**Results:**

In our cohort, 961 patients in the OR group and 1098 patients in the LPEC group were analyzed retrospectively. Mean operative time was significantly shorter in the LPEC group (22.3 ± 3.5 min) than in the OR group (27.8 ± 5.9 min) for bilateral hernia repair (p < 0.001). Postoperative recurrence was 1.3% (13/1035) in the OR group and 0.5% (6/1182) in the LPEC group (p = 0.056). Iatrogenic cryptorchidism occurred statistically more frequently in the OR group than in the LPEC group (0.4% vs. 0%, p = 0.013). In addition, the incidence of MCIH was 3.7% (33/887) in the OR group and 0.3% (3/1014) in the LPEC group (p < 0.01).

**Conclusion:**

Comparing to open technique, laparoscope-assisted single-needle LPEC provides a simple and effective option for pediatric inguinal hernia/hydrocele repair with excellent outcomes, a low incidence of recurrence, and reduced MCIH.

## Introduction

Inguinal hernias and hydroceles are common diseases in pediatric surgery, occurring in 5% of all neonates and almost 10% of premature newborns [1]. Traditionally, inguinal hernia repair is performed with standard open repair (OR) via an inguinal crease incision. Few complications occur, recurrence rates in children are lower than in adults, ranging from 0.5 to 4% [2]. Recently, laparoscopic procedures have become increasingly popular [3]. Normally used minimal access techniques include the transabdominal approach with conventional suturing [4], and the percutaneous approach with different variations, such as single-incision laparoscopic percutaneous extraperitoneal closure (LPEC) [5], percutaneous internal ring suturing (PIRS) [6], or grasping forceps assisted maneuver [7]. The advantages have been reported in laparoscopic repair for children including desirable visual exposure, minimal dissection, less complications, comparable recurrence rate, reduced the risk of metachronous hernia, and improved cosmetic results compared with the traditional open repair [4].

Such minimally access techniques for hernia repair have a steep learning curve [8, 9], which is mainly due to the surgeon’s experience in early-phase and thus contribute to higher rates of recurrence [10, 11]. However, recurrence rates following laparoscopic repair in some series had approximated that of the open repair, ranging from 0.7 to 4.5% [12]. Some comparative studies have also been reported with equal outcomes between laparoscopic hernia repair and conventional methods [13, 14]. Metanalysis based upon data from randomized controlled trails have suggested that recurrence rates between laparoscopic and open repair are similar [15, 16]. In the present study, we described a modification of the surgical technique by using a single needle with laparoscope assistance to accomplish laparoscopic hernia repair. This modification of the LEPC technique intends to devoid of the additional laparoscopic ports and grasping forceps, while via a long tailor-made needle. We hypothesized that the modified LEPC repair of inguinal hernia was better than OR in term of reduced recurrent rate and incidence of metachronous contralateral inguinal hernia(MCIH).

The aim of this study was to compare outcomes of the modified laparoscope-assisted single-needle LPEC versus OR of pediatric hernias/hydrocele from a single institution.

## Materials and methods

This was a retrospective cohort study, including 2059 patients who underwent inguinal hernia repair between 2014 and 2019 in a single institution. The protocol was approved for clinical study by the Ethical Research Committee of Shanghai Children’s Medical Center. The study complied with the Declaration of Helsinki (as revised in 2013) and a written informed parental or guardian consent was obtained. After ethics approval was obtained, medical data of patients who underwent open repair or laparoscope-assisted single-needle LPEC were reviewed. The diagnosis was made by the patient’s medical history, physical examination, ultrasonography, or confirmed by visualization of the laparoscope during surgery. Inclusion criteria were patients aged 0 to 10 years of age, with congenital inguinal hernia or hydrocele. Exclusion criteria: recurrent hernia, complicated hernia, hernia with undescended testis, contraindications for laparoscopy. Demographics, preoperative clinical presentations, and follow-up information were reviewed and analyzed. The choice of the operative method was made on the discretion of the surgeon. The primary outcome measure that was used for comparison was recurrence rate. Secondary outcome measures were: duration of surgery, intraoperative findings (i.e. injury of spermatic cord or vessels, bleeding), postoperative complications (i.e. bleeding/hematoma, wound infection, iatrogenic ascent of testis and testicular atrophy), and incidence of MCIH. Routine follow-up was performed regularly at 1 week for wound healing assessment, and 6, 12, and 24 months postoperatively to evaluate possible complications, in addition to testicular size and position for male patients. We also informed parents to revisit our outpatient clinic if they had any complaints after the regular follow-up period. Telephone interviews were performed for some patients at the 12- and 24-month time points.

### Surgical procedures

#### Open repair

Open hernia ligation was performed by making a 1–3 cm inguinal skin crease incision on the affected side. After the Scarpa’s fascia was incised and the inguinal canal was opened, the spermatic cord structures were scrupulously separated from the hernia sac, and the hernia sac was divided and ligated highly, and then reduced.

#### Laparoscope-assisted single-needle LPEC

**Fig. 1:**
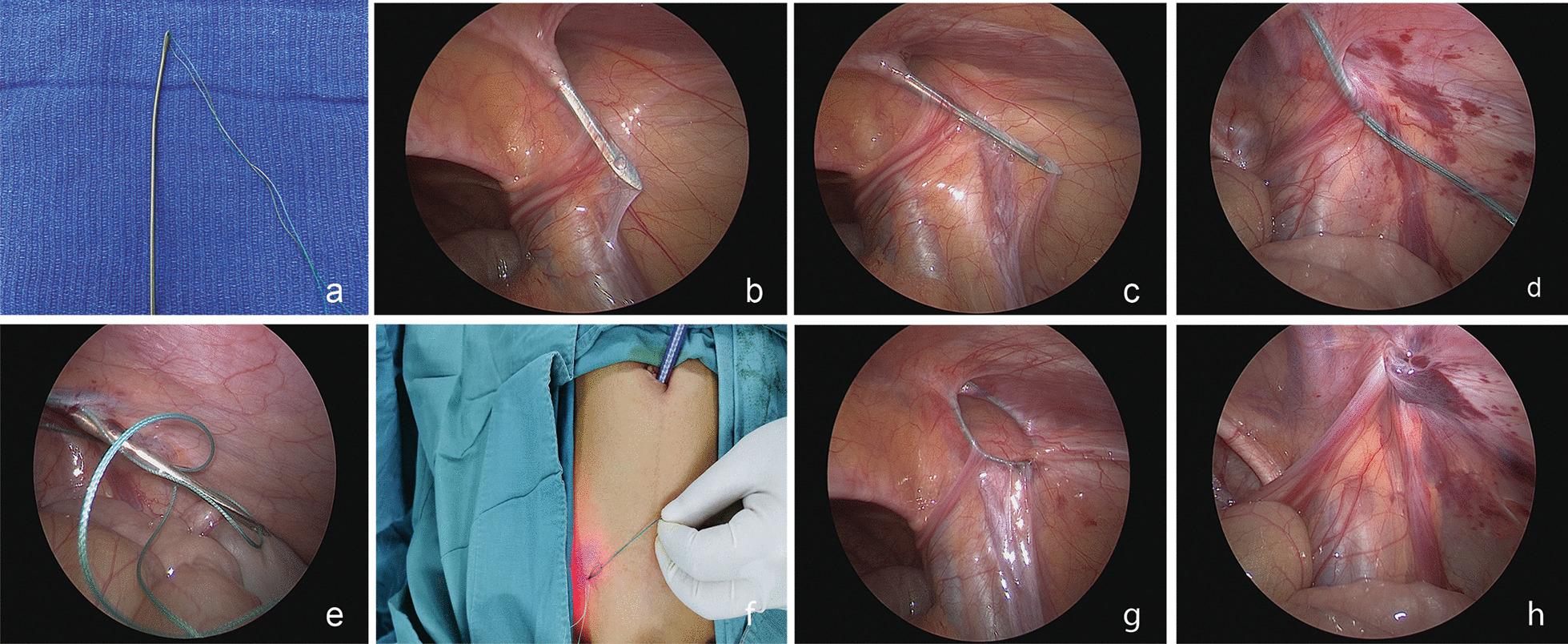
LPEC procedure in right side inguinal hernia for a male patient. **a** Needle tips with eyelet for passing a 2-0 polyester suture; **b**–**d** Medial aspect dissection of the internal ring by negotiating over the vas and vessels with the needle, the first suture loop was left in the abdomen with the assistance of camera feeding the loop; **e**–**g** Lateral advancing to feed the previous loop and the first loop used as a lasso by pulling the extracorporeal end to create purse ring around the internal ring; **h** Ligation of the internal ring

Briefly, all patients were placed in the supine position, and surgery was performed under general anesthesia with tracheal intubation. After pneumoperitoneum was established, a 2 mm stab incision overlying the internal ring on affected side was made, and a 2-0 polyester suture (ETHIBOND EXCEL™, ETHICON, Somerville, NJ) was passed through the eyelet at the tip of the needle (20 cm length) (Fig. 1a). Then, the suture was passed along the medial aspect of the internal ring, traversing over the vas deferens and spermatic vessels. The needle was brought in intraperitoneally, and the suture was left behind with the assistance of the laparoscope to go through the loop, creating the first wire loop in the cavity (Fig. 1b–d). Another suture was then made along the lateral side of the internal ring in a similar fashion. The preloaded loop with the needle was fed through the first loop and left in the abdomen with the assistance of the laparoscope, as previously described. The needle was removed totally, and the first loop acted as a lasso by pulling the extra ends of the suture that created the purse-string circumferentially around the internal ring (Fig. 1e–g). Double ligation was then performed extracorporeally with care to ensure that the skin was free of the knot. Testis should be pulled downward softly and kept in a proper position in the scrotum. Fluid and air were to be evacuated manually from the scrotum or labia or aspirated by a needle with a syringe. A second look was performed to ensure complete ligation of the hernia (Fig. 1h). When asymptomatic contralateral internal ring was confirmed of patency, prophylactic surgery was performed, the same process then repeated.

### Statistical analysis

Statistical analysis was performed using SPSS software (SPSS 22.0, Chicago, Illinois). Descriptive data are presented as mean, standard deviations, median and the interquartile range values of the variables. Data distributions were evaluated by the Kolmogorov–Smirnov test. Normally distributed descriptive variables were compared using the Student-t test. When distributions were not normal, Mann–Whitney U tests were performed when comparing in two groups. Categorical data were mainly analyzed using the Chi-square test or Fisher’s exact test. A *p*-value was considered statistically significant when less than 0.05.

## Results

In our cohort, 961 patients in the OR group and 1098 patients in the LPEC group were analyzed retrospectively. All patient characteristics are presented in Table 1. The median age at operation was 20.5 (IQR 15.7; 65.3) months in the OR group and 23.5 (IQR 14.1; 60.3) months in the LPEC group (*p* = 0.772), and median body weight at operation was 13.5 (IQR 10.1; 23.9) vs. 15.9 (IQR 11.8; 27.7) kg (*p* = 0.682), respectively. There was no statistically significant difference in patient age or weight between the two groups.

**Table 1:** Preoperative patients characteristics in groups

	OR	LPEC	*p* value
No. of patients	961	1098	0.274
Male	918 (95.5%)	1061(96.6%)	
Female	43 (4.5%)	37 (3.4%)	
Age (months, median, and [IQR])	20.5 [15.7; 65.3]	23.5 [14.1; 60.3]	0.772
Weight (kg, median, and [IQR])	13.5 [10.1; 23.9]	15.9 [11.8; 27.7]	0.682
Laterals			0.598
Right	530 (55.3%)	632 (57.5%)	
Left	357 (37.1%)	382 (34.8%)	
Bilateral	74 (7.7%)	84 (7.7%)	
Mean follow-up (months, SD)	18.3 ± 11.6	16.7 ± 15.7	0.587

*IQR*  interquartile range

**Table 2:** Outcomes of procedure

	OR (n = 961)	LPEC (n = 1098)	*p* value
Intraoperative laterals			< 0.01*
Unilateral	887 (92.3%)	619 (56.4%)	
Bilateral	74 (7.7%)	479 (43.6%)	
lateral ligation	1035	1577	
Intraoperative cPPV	–	39% (396 /1014)	
Surgical time (min, SD)			
Unilateral	15.4 ± 6.1	17.4 ± 4.7	0.178
Bilateral	27.8 ± 5.9	22.3 ± 3.5	< 0.01*

*cPPV * contralateral patency processus vaginalis

* *p* < 0.05 is statistically significant

Of all 2059 patients, a total of 1035 hernia rings (887 unilateral and 74 bilateral) were ligated in the 961 patients in the OR group (Table 2). However, in the LPEC group, 1014 of 1098 patients were clinically diagnosed with unilateral hernia (632 right-sided and 382 left-sided inguinal hernia), and 39% (396/1014) of them were confirmed to have asymptomatic contralateral PPV intraoperatively and underwent prophylactic surgery. Hence, in the LPEC group, 619 of 1098 patients underwent unilateral surgery and 479 of 1098 underwent bilateral surgery. A total of 1577 internal inguinal rings (619 unilateral and 479 bilateral, including 396 contralateral PPVs) were ligated in the 1098 patients in the LPEC group (Table 2). Operative time was compared between the two groups, mean operative time for unilateral surgery in the OR and LPEC groups were 15.4 ± 6.1 min and 17.4 ± 4.7 min, respectively. The difference was not statistically significant in unilateral repair (*p* = 0.178), while the operative time was significantly shorter in the LPEC group (22.3 ± 3.5 min) than in the OR group (27.8 ± 5.9 min) for bilateral repair (*p* < 0.01, Table 2).

A comparison of complications between the OR and LPEC groups are shown in Table 3. All LPEC administrations were performed without open conversion or requiring additional skin incision. For 8 male patients whose hernia contained omentum adhesive to the hernia sac, another 3 mm forceps were introduced through the extended infraumbilical incision to retract to the abdomen. There were no injuries observed to any intra-abdominal organs except in cases of bleeding or hematoma. Inguinal bleeding or hematoma occurred more frequently in the OR group (1.8%; 19/1035) than in the LPEC group (0.1%; 2/1577) (*p* < 0.01). All bleeding or hematoma was observed regularly or controlled by external compression at the groin site. In the OR group, wound infections were recorded in 11 cases (1.1%), whereas in the LPEC group, only 3 cases (0.3%) developed. There were no statistically significant differences between the two groups regarding wound infection that needed further treatment (*p* = 0.16).

**Table 3:** Comparison of the complications in groups

				OR (n = 961)		LPEC (n = 1098)		*p* value		
Bleeding/hematoma				1.8% (19/1035)		0.1% (2/1577)		< 0.01*		
Wound infection				1.1% (11/961)		0.3% (3/1098)		0.16		
Iatrogenic ascending testis				0.4% (4/984)		0/1525		0.013*		
Testicular atrophy				0.2% (2/984)		0/1525		0.078		
Recurrence				1.3% (13/1035)		0.5% (6/1182)		0.056		
MCIH				3.7% (33/887)		0.3% (3/1014)		< 0.01*		

*MCIH* Metachronous Contralateral Inguinal Hernia

* *p* < 0.05 is statistically significant

The mean follow-up period was 18.3 ± 11.6 months in the OR group and 16.7 ± 15.7 months in the LPEC group (*p* = 0.587). The total follow-up rates were 91.3% and 84.7% at 12 and 24 months, respectively. During this period, iatrogenic cryptorchidism occurred statistically more frequently in the OR group (0.4%; 4/984) than in the LPEC group (0%; 0/1525) (*p* = 0.013). Testicular atrophy was found in 2 patients in the OR group (0.2%; 2/984) and 0% in the LPEC group (0/1525; *p* = 0.078). In addition, postoperative recurrence was 1.3% (13/1035) in the OR group and 0.5% (6/1182) in the LPEC group (*p* = 0.056) (Table 3). Intraoperatively, contralateral patency processus vaginalis (PPV) was detected in 396 of 1014 (39%) patients who were diagnosed with a clinically unilateral presentation. The contralateral PPV was confirmed by the evidence of insufflation in the groin through the PPV to the scrotum or labia, and all these patients underwent prophylactic surgery. As prophylactic surgery was related to MCIH, MCIH was analyzed in patients who had a clinically unilateral inguinal hernia. The incidence of MCIH was 3.7% (33/887) in the OR group and 0.3% (3/1014) in the LPEC group (*p* < 0.01) (Table 3). All MCIH and recurrence were re-operated laparoscopically. All patients in this study were discharged in 1 or 2 days.

## Discussion

The standard treatment for inguinal hernia is surgical ligation of PPV through inguinal incision, evolving significantly in the last 50 years. Open inguinal hernia repair is an excellent method for children [2]. However, it has the potential risk of injury of the vas deferens and spermatic vessels, iatrogenic ascending testis or testicular atrophy and recurrence. As the techniques develop promptly, the laparoscopic approach has been introduced in the past three decades, with grossly reported in the literature demonstrating safety and efficacy in children [17–20]. Traditionally, multiple trocars placement should be placed for laparoscopic hernia repair [21], which is associated with extended operative time due to transabdominal suturing, and increased postoperative pain, more complications (wound infection, incisional hernia). Further, technical refinements have led to the emergence of newer techniques [22–24], such as subcutaneously and percutaneously. Spinal needle [8], two-hooked device [25], needle-like apparatus [26] or others have been used for closure of PPV. Pogorelić et al. [6] reported the excellent outcomes with PIRS technique, which involves only a single umbilical port using for introduction of a laparoscope and a hollow spinal needle for percutaneous closure of hernia.

Our technique is a tiny modification to the established approaches of percutaneous ligation of the internal ring with the assistance of a laparoscope (Fig. 1). Technically, this modification is similar in that of PIRS [6], it is unique in that a long eye-letted needle is used to maneuver around the internal ring percutaneously instead of a suturing in other similar reports. No manipulation is required as the dissection and ligation occur as high as possible. Excellent visual exposure confirms that only peritoneum is encircled by the suture and that the vessels and vas deferens are not compromised. The risk of injury to these structures is also minimized as the needle is superiorly avoided to these cord structures. In our cohort, no iatrogenic ascending testis, testicular atrophy have been detected to date. However, long-term follow-up is required to evaluate the difference in testicular complications between these two approaches, but early results in our cohort are promising.

The main complication of inguinal hernia repair is recurrence [12]. The factors affecting recurrence seem to be failure to ligate the hernia sac high enough at the internal ring [27], operative trauma leading to injury of the floor of the inguinal canal, failure to close the internal ring tightly, and postoperative hematoma and wound infection [28]. Some authors hypothesize that these possible causes of recurrence can be avoided by the laparoscopic technique [29]. We observed a recurrence rate of 0.5% after laparoscopic hernia repair in children. This is similar to the recurrence rate published in the literature where recurrence rates following laparoscopic repair roughly between 0.7 and 4.5% are described [12]. Our recurrences mainly occurred during the learning curve for laparoscopic repair, and we observed less recurrences after familiar with the technique. However, when comparing the recurrence rate of inguinal hernia after laparoscopic and open repair, no significant difference was found in our cohort. Nevertheless, it must be kept in mind that in this study, shorter follow-up intervals were noted after laparoscopic inguinal hernia repair when compared with open procedure.

Another issue is the identification of contralateral PPV, which has been found during surgery is reported to occur with a range of 20–60% [30]. In this study, contralateral PPV was confirming in 39%, after which prophylactic ligation was performed, therefore, the incidence rate of MCIH was reduced from 3.7% in conventional open repair to 0.3% in LPEC. The administration of contralateral PPV remains controversial [30], and there is also a concern for overtreatment [31]. Despite the relatively high prevalence of contralateral PPV in unilateral inguinal hernia, some pediatric surgeons argue that a PPV at exploration may not necessarily mean possible development of a hernia at a later age [32]. Meanwhile, the follow-up period has been very limited to the age period in most patients. However, another prospective cohort study indicates that asymptomatic PPV is a risk factor for developing inguinal hernia in adults. So, to the best of the author’s knowledge, contralateral PPV should be viewed as a possible factor in developing inguinal hernia beyond the pediatric period [33]. That is the reason the authors perform the routine contralateral prophylactic ligation for asymptomatic PPV. A prospective, randomized study with long-term follow-up is required to confirm this in the future.

Some may suspect that the learning curve of the procedure might affect the outcome of the operation [9]. However, this laparoscopic approach is a simple manipulation with a single port, without the intracorporeal suturing. The training curve is steep [10], which 10 to 20 cases are sufficient to gain expertise. Actually, two surgeons in this study were trained to perform the procedure with basic proficiency in laparoscopy, and they were easily adhered to the implementation of a minimally invasive surgery to perform the inguinal hernia repair.

However, this study also has limitations. This study is a short-term retrospective study conducted at a single institution. As the choice of the operative method was relied on the discretion of the surgeon, we are not able to rule out selection bias. Patient selection is also an important consideration. Although we include the adolescent group during inclusive period, we have difficulties collecting enough date for statistical analysis. Concerns about MCIH as well as testicular ascending or atrophy may need long-term follow-up for evaluation. Continued follow-up and accurate reports of recurrence are important to find the differences. A randomized, prospective study with long-term follow-up is necessary in the future.

## Conclusion

LPEC provides a minimally invasive approach with comparable results from the perspective of recurrent rates and testicular complications based on short-term outcomes. It is also argued that prophylactic surgery for contralateral PPV is beneficial for preventing MCIH without increased complication compared to the conventional open approach. Based on these data, it is recommended that laparoscope-assisted single-needle LPEC be used as an option to treat inguinal hernia in children.

## Data Availability

The datasets used and/or analyzed during the current study are available from the corresponding author on reasonable request.

## Abbreviations

OR: Open repair
LPEC: Laparoscopic percutaneous extraperitoneal closure
PPV: Patency processus vaginalis
MCIH: Metachronous contralateral inguinal hernia
PIRS: Percutaneous internal ring suturing
IQR: Interquartile range
